# Verotoxin-1 Treatment or Manipulation of its Receptor Globotriaosylceramide (Gb3) for Reversal of Multidrug Resistance to Cancer Chemotherapy

**DOI:** 10.3390/toxins2102467

**Published:** 2010-10-25

**Authors:** Parviz Behnam-Motlagh, Andreas Tyler, Kjell Grankvist, Anders Johansson

**Affiliations:** 1 Department of Medical Biosciences, Umeå University, S-901 85 Umea, Sweden; Email: parviz.behnam@medbio.umu.se (P.B.-M.); andreas.tyler@medbio.umu.se (A.T); kjell.grankvist@medbio.umu.se (K.G.); 2 Department of Odontology, Umeå University, S-901 85 Umea, Sweden

**Keywords:** apoptosis, cancer, Gb3, verotoxin-1, multi-drug resistance, *MDR1*, P-gp

## Abstract

A major problem with anti-cancer drug treatment is the development of acquired multidrug resistance (MDR) of the tumor cells. Verotoxin-1 (VT-1) exerts its cytotoxicity by targeting the globotriaosylceramide membrane receptor (Gb3), a glycolipid associated with multidrug resistance. Gb3 is overexpressed in many human tumors and tumor cell lines with inherent or acquired MDR. Gb3 is co-expressed and interplays with the membrane efflux transporter P-gp encoded by the *MDR1* gene. P-gp could act as a lipid flippase and stimulate Gb3 induction when tumor cells are exposed to cancer chemotherapy. Recent work has shown that apoptosis and inherent or acquired multidrug resistance in Gb3-expressing tumors could be affected by VT-1 holotoxin, a sub-toxic concentration of the holotoxin concomitant with chemotherapy or its Gb3-binding B-subunit coupled to cytotoxic or immunomodulatory drug, as well as chemical manipulation of Gb3 expression. The interplay between Gb3 and P-gp thus gives a possible physiological approach to augment the chemotherapeutic effect in multidrug resistant tumors.

## 1. Shiga Toxins

The Shiga toxin family, a group of related exotoxins, includes Shiga toxin from *Shigella dysenteriae* and Shiga toxins such as verotoxin-1 (VT-1, Shiga-like toxin-1) produced by pathogenic strains of *Escherichia coli* [1,2,3].

VT-1 consists of one A and five B subunits. The B subunit binds to the neutral glycosphingolipid cell surface receptor globotriaosylceramide (Gb3) [4,5] and is endocytosed. The toxin then follows the retrograde pathway to the endoplasmic reticulum where the A-subunit is translocated to the cytosol and inhibits protein synthesis [6,7,8,9,10,11,12].

VT-1 also induces apoptosis through sequential activation of caspases, leading to nuclear changes, such as chromatin condensation and DNA fragmentation. VT-1-induced apoptosis in monocytic THP1 cells requires retrograde transport through the Golgi apparatus to the ER and the activation of caspase-3, the executioner caspase [13]. Similar apoptotic signaling pathways are triggered by Shiga toxins in different cell lines. 

VT-1 induces a prominent ribotoxic stress signaling response leading to disrupted ribosomal RNA (rRNA) functions, protein synthesis inhibition and altered mitogen-activated protein kinase (MAPK) pathway signaling [14]. We found that MKK3/6 and JNK was phosphorylated after cisplatin treatment in the cisplatin-sensitive malignant pleural mesothelioma (MPM) cells, but not in the corresponding sub-lines with acquired-cisplatin resistance. VT-1 induced phosphorylation of MKK3/6, which was enhanced when VT-1 was combined with cisplatin [15]. MKK3/6 is known to activate P38 [16,17]. P38, as well as JNK, has been shown to promote apoptosis in response to cellular stress [18]. Treatment of cells with chemical inhibitors or siRNA targeting P38 was recently shown to specifically inhibit VT-1 transport to the Golgi apparatus complex and reduce VT-1 toxicity [19], and VT-1 prolonged JNK and P38 MAPK activation of macrophage-like cells [20]. We have previously demonstrated JNK phosphorylation in response to VT-1 treatment also in glioma and breast cancer cell lines [21,22].

Apoptosis induced by VT-1 was associated with enhanced expression of the pro-apoptotic protein Bax [23] and overexpression of Bcl-2 protects cells against VT-1-induced cell death [24]. Shiga toxins also inhibit the expression of the anti-apoptotic Bcl-2 family member Mcl-1 [25]. Interestingly, acquisition of cisplatin resistance in MPM cells decreased cisplatin activation of the proapoptotic proteins of the Bcl-2 family of proteins [26] and increased the expression of apoptosis inhibitor proteins [27].

## 2. Glycosphingolipids and Globotriasosylceramide (Gb3)

Glycosphingolipids (GSLs) are components of all vertebrate cells and play a fundamental role during development and cell differentiation [28]. GSLs are involved in cellular growth [29], signal transduction [30] and cell-cell interaction [31]. GSL profiling indicates that neutral globo series GSLs (including Gb3) have important roles in mediating MDR1 transactivation and expression [32]. Deletion of Gb_3_ synthase needed for Gb3 synthesis renders mice completely resistant to VT-1 and VT-2 [33] and GSLs are the only functional VT-1 receptors [34].

GSLs in cells are clustered and assembled with specific membrane proteins and signal transducers to form GSL-enriched microdomains or lipid rafts [35,36,37]. Rafts are rich in GSLs, cholesterol, lipid-modified- and transmembrane proteins [38]. The length of the fatty acyl chain of Gb3 influences its receptor function, intracellular sorting and retro-translocation of VT-1 to the cytosol [39,40]. Binding of VT-1 B-subunit with clustered raft-localized Gb3 receptors [41] is a requirement for the retrograde transport [42] and for a cytotoxic effect in the ER [43]. For cells with Gb_3_ present in the non-raft plasma membrane fraction, the toxin receptor complex is internalized and trafficked to lysosomes where the toxin is degraded, leading to VT-1 resistant cells. [44]. Furthermore, VT-1 B subunit binding to Gb3 induces lipid reorganization of the cell membrane leading to enhancement of VT-1 uptake into the cell [45].

Gb_3_ membrane organization also plays a central role in determining *in vivo* sensitivity to verotoxin-induced glomerular pathology of hemolytic uremia syndrome (HUS). Gb_3_ is distributed throughout the human nephron but only the Gb3 of the glomeruli is localized to lipid rafts making glomeruli sensitive to the cytopathology of systemic VT-1. The membrane organization of the glycosphingolipid receptor is the main discriminator for pathology *in vivo* [34,46].

The expression and metabolism of cell surface glycolipids is changed during oncogenic transformation and altered glycosylation patterns affect tumor invasion and metastasis [37]. Gb_3_ is expressed in several human malignancies including breast cancer [22] and testicular carcinoma [47]. Gb_3_ expression has been detected in lymphoma [48] and in various solid tumors [49]. Gb_3_ expression in colorectal cancer correlates with invasiveness and metastatic potential [50]. Elevated levels of Gb3 have also been seen in drug-resistant cancers and cell lines and a functional interplay between membrane Gb3 and MDR1 has been suggested [51,52]. These findings suggest that the Gb3-binding specificity of VT-1 could be used to target tumors in the receptive cancer cells.

## 3. Multidrug Resistance to Cancer Chemotherapy

Poor response to cancer chemotherapy is usually due to drug resistance [53,54]. In breast cancer alone, nearly 50% of patients demonstrate primary and/or secondary resistance to doxorubicin [55]. 

Tumor overexpression of the membrane efflux transporter P-glycoprotein (P-gp) is a common alteration in drug resistance [53,54,56]. P-gp, encoded by the *MDR1* gene [57], was the first ABC protein demonstrated to confer resistance to cancer chemotherapeutics [58,59]. Other transporter proteins such as multidrug resistance protein (MRP1) and breast cancer resistance protein (BCRP) have also been described. P-gp plays roles in the absorption, distribution and excretion of compounds in normal tissues. Overexpression of MDR1 in tumors results in active efflux of several types of anticancer agents. P-gp is expressed by many types of primary solid tumors such as breast, colon, renal, and ovarian cancers, as well as hematological malignancies such as acute myeloid leukemia and non-Hodgkin's lymphoma [60]. 

Exposure to chemotherapy can up-regulate tumor P-gp expression, which occurs in acquired drug resistance [61] and severely limits the success of chemotherapy [54,62]. In small cell lung cancer, acquired resistance to multiple drugs is responsible for a chemotherapeutic cure rate below 10% [63]. In breast cancer, 55% of the tumors expressed P-gp 55% before and 100% after chemotherapy [64]. MDR1 inhibitors have been clinically tested in order to block drug efflux. Specific modulators or inhibitors such as GG918 and LY335979 have overcome the toxic adverse effects noted in first generation modulators but still have minor effect when co-administrated with chemotherapeutics in trials [65,66] in part due to *MDR1* polymorphisms [32].

## 4. Globotriasosylceramide (Gb3) and MDR1 Expression

Little is known about the molecular mechanism underlying *MDR1* overexpression and how it interacts with other genes to impart drug-resistance. Overexpression of glucosylceramide synthase (GCS), the first enzyme of GSL synthesis, can result in multidrug resistance. Many cells expressing *MDR1* show elevated levels of glucosylceramide (GlcCer) [67,68], and inhibitors of GCS kill MDR cells [69]. MDR1 can translocate glucosylceramide into the Golgi apparatus for neutral GSL synthesis, including Gb3. P-gp has been proposed as a Golgi glucosylceramide flippase that enhances neutral GSL synthesis as transfection of *MDR1* increases, and inhibition of P-gp decreases neutral GSL biosynthesis in cells [70]. GCS up-regulates *MDR1* expression and modulates drug resistance of cancer.

Partial MDR1 and Gb_3_ cell surface co-localization has been observed and inhibition of GSL biosynthesis depletes cell surface MDR1. MDR1 may therefore interact with Gb_3_. A significant fraction of surface MDR1 is not co-localized with Gb_3_, and could therefore be VT-1-insensitive. MDR1 can be expressed in cells lacking Gb_3_. However, drug-resistant metastatic ovarian tumor cells have a particularly high Gb_3_ content [49] and Gb_3_ is highly expressed in metastatic colon carcinoma [50]. 

The water-soluble Gb_3_ mimic adamantylGb_3_, but not other GSL analogs, reversed MDR1-MDCK cell drug resistance [51]. Verotoxin-mediated Gb_3_ endocytosis also up-regulated total MDR1 and inhibited drug efflux [71].

The Gb3 content, which is regulated by the expression of Gb3 synthase, was demonstrated to determine the sensitivity of HeLa cells toward VT-1 [72]. We recently demonstrated extensive variability in breast cancer cell lines for apoptosis induction by VT-1. Sensitivity was correlated with Gb3 expression, and use of the drug PPMP, which down-regulates glucosylceramide production, inhibited VT-1-mediated apoptosis [22]. Verotoxin-1 has shown efficacy against meningioma, astrocytoma and renal tumor xenografts in mice [73,74,75].

## 5. Tumor Targeting

The possibility that VT-1 through the A-subunit could cause protein synthesis inhibition and induce apoptosis in normal cells constitutes a concern for the use of the holotoxin as an anticancer agent. The non-toxic VT-1 B subunit is stable at extreme pH, resists proteases, crosses tissue barriers, distributes in the organism and generally resists extra- and intracellular inactivation [76]. The receptor selectivity of the B subunit has therefore been used to couple it to cytotoxic compounds such as the topoisomerase I inhibitor SN38 [77] or induce an immune response [78] with preferential effects on cancer cells. 

Of primary cultures of gastrointestinal tumors, 80% were found to bind the VT-1 B subunit and could be detected on tumor cells after five days. The stable association of VT-1 B subunit with cells might be a useful property for diagnostic or therapeutic delivery strategies. This subunit has little immunologic properties [79] and is well tolerated in a mouse model [44]. 

An apparent treatment possibility to reverse MDR is to inhibit GSL biosynthesis by inhibiting GCS or Gb3 synthase enzyme expression and/or activity, or use Gb3 mimics like adamantylGb_3_ [51]. 

**Figure 1:**
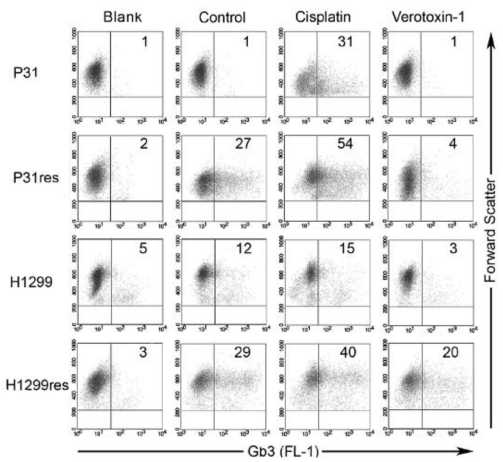
Flow cytometry analysis of membrane bound Gb3 expression by cultured cisplatin-resistant (res) and non-resistant malignant pleural mesothelioma cells (P31) and non-small cell lung cancer cells (H1299) not incubated with and cells incubated for 72  h with 5  mg  L^–1^ cisplatin or 0.1  *μ*g L^–1^ VT-1, respectively. The percentage of Gb3-expressing cells is noted in the right quadrant in each dot plot. Blank shows unspecific secondary antibody binding, whereas control shows cells not incubated with either cisplatin or VT-1. Cisplatin resulted in further enhanced Gb3 expression, while VT-1 treatment resulted in a complete eradication of the Gb3 expressing cell population [15].

The treatment obstacle of acquired-cisplatin resistance in malignant plural mesothelioma (MPM) and other cancers makes it necessary to find new strategies to overcome resistance. We showed that cisplatin can up-regulate Gb3 expression in MPM and NSCLC cells and thus sensitize the cells to VT-1-induced cytotoxicity (Figure 1). The increased proportion of Gb3-expressing cells after cisplatin treatment suggests that cisplatin induces Gb3 expression in cancer cells, that cisplatin preferentially eradicates cell with low Gb3 expression and that Gb3 expression is linked to acquired cisplatin-resistance [15]. We could also correlate increased expression of Gb3 in cisplatin-resistant MPM and NSCLC cells to increased expression of MDR1/PgP. PPMP reduced Gb3 expression in resistant sub-line cells and particularly of the Gb3-expressing fraction that was induced when the mother cell line was made cisplatin-resistant. A strong super-additive effect of combined cisplatin and a sub-toxic concentration of VT-1 in cisplatin-resistant malignant pleural mesothelioma cells were observed, indicating a new potential and urgently needed clinical treatment approach [15].

The MAPK pathway is involved in proapoptotic signaling of VT-1 in stressed cell systems and the pathway is also involved in cisplatin-induced apoptosis and induced cisplatin resistance [15,80]. Targeting the MAPK signaling pathway could, therefore, be an additional way to reduce cisplatin-induced tumor cells resistance. 

The partial cell surface co-localization of Gb3/MDR1, the modulation of MDR1 cell surface expression by GSL and the possibility to inhibit MDR1 expression by VT-1/VT-1 B-subunit, all indicate a functional link between Gb3 and MDR1. Targeting the physiological regulation of MDR1 could be an efficient way not only to prevent the development of drug resistance during cancer chemotherapy but also to reverse inherent and acquired drug resistance of cancers.
